# *Drosophila* Spastin Regulates Synaptic Microtubule Networks and Is Required for Normal Motor Function

**DOI:** 10.1371/journal.pbio.0020429

**Published:** 2004-11-30

**Authors:** Nina Tang Sherwood, Qi Sun, Mingshan Xue, Bing Zhang, Kai Zinn

**Affiliations:** **1**Broad Center, Division of Biology, California Institute of TechnologyPasadena, CaliforniaUnited States of America; **2**Section of Neurobiology, University of TexasAustin, TexasUnited States of America

## Abstract

The most common form of human autosomal dominant hereditary spastic paraplegia (AD-HSP) is caused by mutations in the *SPG4 (spastin)* gene, which encodes an AAA ATPase closely related in sequence to the microtubule-severing protein Katanin. Patients with AD-HSP exhibit degeneration of the distal regions of the longest axons in the spinal cord. Loss-of-function mutations in the Drosophila spastin gene produce larval neuromuscular junction (NMJ) phenotypes. NMJ synaptic boutons in *spastin* mutants are more numerous and more clustered than in wild-type, and transmitter release is impaired. *spastin*-null adult flies have severe movement defects. They do not fly or jump, they climb poorly, and they have short lifespans. *spastin* hypomorphs have weaker behavioral phenotypes. Overexpression of Spastin erases the muscle microtubule network. This gain-of-function phenotype is consistent with the hypothesis that Spastin has microtubule-severing activity, and implies that *spastin* loss-of-function mutants should have an increased number of microtubules. Surprisingly, however, we observed the opposite phenotype: in *spastin*-null mutants, there are fewer microtubule bundles within the NMJ, especially in its distal boutons. The *Drosophila* NMJ is a glutamatergic synapse that resembles excitatory synapses in the mammalian spinal cord, so the reduction of organized presynaptic microtubules that we observe in *spastin* mutants may be relevant to an understanding of human Spastin's role in maintenance of axon terminals in the spinal cord.

## Abstract 2

Kai Zinn and colleagues use loss- and gain-of function mutations to study the Drosophila homologue of a gene mutated in human autosomal dominant hereditary spastic paraplegia

## Introduction

“Pure” autosomal dominant hereditary spastic paraplegia (AD-HSP) is an inherited disease characterized by bilateral spasticity in the absence of other phenotypes (reviewed in Fink 2003; Reid 2003). Afflicted patients experience difficulty in walking and have a distinctive gait. Degeneration of the lateral corticospinal tracts, which contain the axons of cortical neurons that innervate primary limb motoneurons, is observed in the lumbar regions of the spinal cord in patients with AD-HSP. The distal segments of long dorsal root ganglion axons also display degeneration. No evidence is seen for cell death or for primary myelination defects, and the axons of primary motor neurons do not degenerate (Maia and Behan 1974; Wharton et al. 2003). AD-HSP thus appears to selectively affect the distal regions of the longest axons within the spinal cord.

Because pathology is usually confined to long spinal cord axons, it has been suggested that the primary defect in pure AD-HSP is in axonal transport or some other process required for maintenance of axon terminals. Perturbation of anterograde or retrograde axonal transport might selectively affect the longest axons, because they would be most vulnerable to a reduction in efficiency of transport of material to or from their terminals.

About 40% of cases of pure AD-HSP are caused by mutations in the *SPG4* gene, which encodes an AAA ATPase called Spastin (Hazan et al. 1999). AAA ATPases are a large and diverse set of proteins that include an approximately 250–amino acid (aa) conserved domain containing Walker A and B ATP-binding motif sequences (reviewed in Confalonieri and Duguet 1995; Patel and Latterich 1998; Neuwald et al. 1999). They use energy obtained from ATP hydrolysis to catalyze assembly or disassembly of a variety of protein complexes. AAA proteins are involved in many cellular processes, including vesicle trafficking, protein degradation, and microtubule dynamics. Many AAA ATPases form hexameric rings, and it is thought that the ring structures are required for catalytic activity (Vale 2000).

Spastin is a member of the “meiotic” subgroup of AAA ATPases (Frohlich 2001; Frickey and Lupas 2004), which contains proteins involved in vesicle trafficking and microtubule dynamics. The only member of the subgroup whose activities have been biochemically characterized is Katanin-60, which is the catalytic subunit of a microtubule-severing protein (McNally and Vale 1993; Hartman et al. 1998). Katanin-60 and Spastin are homologous only within their AAA domains. However, cell culture studies have provided evidence that Spastin is also involved in microtubule dynamics. Expression of wild-type human Spastin in transfected cell lines and cortical neurons caused disassembly of the microtubule cytoskeleton, while a mutant Spastin lacking catalytic activity colocalized with tubulin (Errico et al. 2002; McDermott et al. 2003).

The mechanisms by which *spastin* mutations produce dominant spasticity phenotypes in humans are controversial. A wide variety of nonsense and missense mutations, but no complete gene deletions, have been found in families with AD-HSP. It has been suggested that dominance arises from haploinsufficiency (Charvin et al. 2003). This model, however, would require that the processes in which Spastin participates are vulnerable to a 50% decrease in its enzymatic activity. Another possibility is that truncated or missense mutant Spastins function as dominant negatives. Hexameric AAA ATPase ring complexes might be especially vulnerable to the presence of nonfunctional (“poison”) subunits that assemble into rings but lack catalytic activity. Consistent with this idea, expression of a mutant Spastin that associates with microtubules but cannot catalyze severing altered organelle distribution in transfected cells (McDermott et al. 2003). In the dominant negative model, AD-HSP might only occur when Spastin activity is eliminated or greatly reduced.

In this study, we describe the phenotypes arising from mutation of the *Drosophila* ortholog of human *spastin*. We initially identified this gene in a gain-of-function screen, in which we found that its overexpression in neurons causes axons in the embryonic central nervous system (CNS) to converge onto the midline (Sun 2000). Overexpression of Spastin in muscles erases their microtubule networks, consistent with the idea that Spastin is a microtubule-severing protein.

We made loss-of-function (LOF) *spastin* mutations, and found that they produce recessive phenotypes affecting the larval neuromuscular system. The *Drosophila* neuromuscular junction (NMJ) uses glutamate as its neurotransmitter and employs ionotropic glutamate receptors homologous to vertebrate AMPA receptors (Schuster et al. 1991; Petersen et al. 1997; Marrus et al. 2004). It is organized into presynaptic boutons that are surrounded by a postsynaptic scaffold, and its synapses exhibit plastic behavior during development. These properties make the fly NMJ a useful genetic model system for the study of glutamatergic synapses in the mammalian brain and spinal cord (Keshishian et al. 1996; Koh et al. 2000).

During the period from larval hatching through the third instar stage, the number of boutons at each NMJ increases by up to 10-fold in order to keep pace with the growth of its muscle target. New boutons are added by a process of budding (Zito et al. 1999). As these boutons mature, their microtubule cytoskeleton is thought to progress through a regulated series of alterations (Roos et al. 2000; Pennetta et al. 2002).

In this paper, we show that synaptic growth and function are altered in *spastin* mutant larval NMJs. Boutons are more numerous than in wild-type larvae, and synaptic transmission is impaired. These changes could result from alterations in synaptic microtubule dynamics, because we find that microtubule bundles are depleted from the distal boutons of NMJs in *spastin*-null mutants. This is surprising, because the fact that Spastin overexpression destroys microtubule networks might lead one to expect that its removal would increase the number of microtubules. Morphological and microtubule phenotypes are seen only for a total gene deletion, indicating that complete loss of Spastin function is required to alter synaptic microtubules in the fly system. The phenotypes we see are quite different from those described in a recently published study of perturbation of *Drosophila* *spastin* using RNAi methods (Trotta et al. 2004). In particular, the changes in synaptic microtubules that occur in *spastin* LOF mutants are opposite to those reported in the RNAi perturbation paper.

*spastin* is not an essential gene, but mutant adults have severely compromised motor behavior. Null mutants cannot fly or jump, they climb slowly, and they often drag their hind legs. While it is intriguing that *spastin* mutant flies display such movement phenotypes, further work will be required to determine whether *Drosophila* can provide a useful organismal model system for human AD-HSP. Nevertheless, insights into the cellular functions of *Drosophila* Spastin obtained from our work should be relevant to an understanding of Spastin's functions in human neurons.

## Results

### The Drosophila spastin Gene

We identified *spastin* in an *“EP”* screen for genes involved in embryonic CNS development. *EPs* are *P* element derivatives with a block of “UAS” sites recognized by the yeast transcription factor GAL4 near one end (Rorth 1996). An *EP* element inserted in the proper orientation upstream of a gene will drive its expression in a cell-specific manner when the insertion line is crossed to the appropriate promoter-*GAL4* “driver” line (Brand and Perrimon 1993). We generated approximately 6,000 new *EP* insertion lines and screened them by crossing to pan-neuronal (*Elav^C155^*) and pan-muscle (*24B*) *GAL4* driver lines (Lin and Goodman 1994; Luo et al. 1994). Those lines for which crosses to either driver generated reduced numbers (<20% of expected) of viable adult progeny containing both the *EP* element and the driver were saved. About 2% of lines (131) exhibited lethality or reduced viability with one of the drivers, and 62 of these were lethal or semilethal with both drivers. The *T32* insertion on the third chromosome conferred complete lethality when crossed to either driver, and produced a neuronal-driver-dependent axonal phenotype (see below).

**Figure 1:**
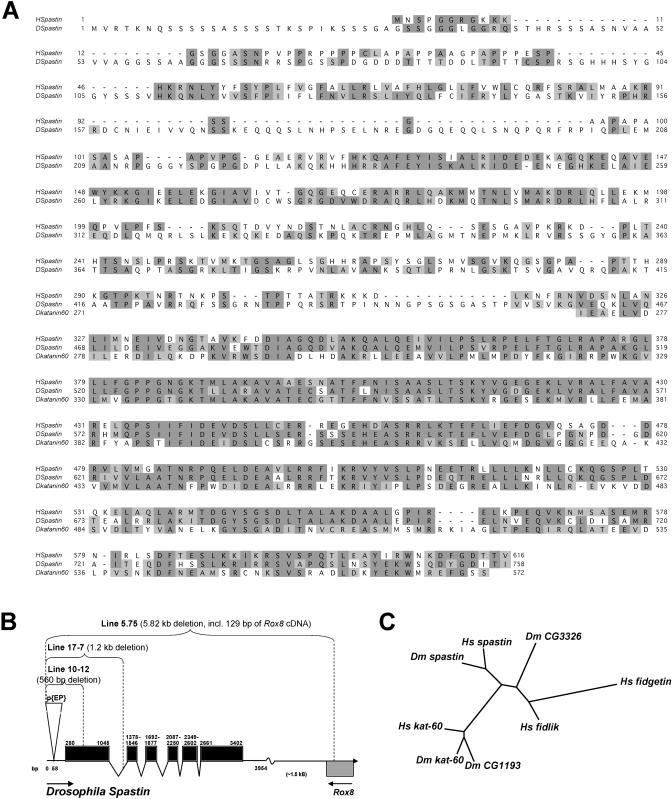
*Drosophila* Spastin: Sequence Alignment and Gene Map (A) Clustal alignment of complete D. melanogaster and H. sapiens Spastin amino acid sequences and the AAA region of *Drosophila* Katanin-60. Identical and similar residues are highlighted in dark and light gray, respectively. (B) Map of the *Drosophila* *spastin* gene, including exons (black boxes) and introns, the position of the *T32* *EP* insertion (nucleotide 58 of the 5′ UTR), and the regions deleted by imprecise excision in lines 10-12, 17-7, and 5.75. The 3′ end of the adjacent *Rox8* gene is also shown. Arrows indicate direction of transcription. (C) Unrooted phylogenetic tree generated by the “neighbor” algorithm, showing relationships between the AAA domains of Spastins and their close relatives in human and fly. Dm CG3326 is the counterpart of the human *fidgetin/fidlik* gene pair, while CG1193 probably encodes a second fly ortholog of human Katanin-60. In the mouse, *fidgetin* mutations produce inner ear defects that cause head-shaking and circling behaviors (Cox et al. 2000).

To identify the gene driven by the *T32* element, we cloned a genomic DNA fragment adjacent to the insertion site and used it to identify a full-length cDNA encoding a 758-aa protein that is a member of the AAA ATPase family (Figure 1A). The *T32* *EP* element is inserted into the 5′ UTR, 222 nucleotides upstream of the predicted ATG start codon (Figure 1B; Sun 2000).

The gene driven by *T32* is orthologous to the human *SPG4* *(spastin)* gene that is mutated in the most common form of AD-HSP (Hazan et al. 1999). Mammalian Spastins are the only proteins that are homologous to both the C-terminal AAA domain and the N-terminal region of fly Spastin (Figure 1A). Spastin exhibits homology to all other AAA proteins only within its AAA domain (approximately aa 460–754 of fly Spastin). There are about 30 AAA proteins encoded in the *Drosophila* genome.

The *Drosophila* Spastin sequence from aa 233 to the C terminus is 49% identical to that of human Spastin (616 aa). The AAA domains of the two proteins are 67% identical. The other region that is conserved between the Spastins (34% identity) corresponds to aa 233–404 of the fly sequence. The same region is also weakly related (26% identity) to human Spartin, the product of the *SPG20* gene mutated in Troyer syndrome, a form of “complicated” HSP (Patel et al. 2002; Ciccarelli et al. 2003). Spartin is not an AAA ATPase. The AAA protein with a known biochemical function that is most closely related to Spastin (41% identity in the AAA domain) is Katanin-60 (Figure 1A and 1C; McNally and Vale 1993; Hartman et al. 1998).

### *Drosophila* Spastin Localizes to the Cytoplasm

Human Spastin is thought to be a cytoplasmic protein expressed in many cell types, based on localization of epitope-tagged proteins expressed in transfected cells, and antibody staining of human tissue and neuronal cell lines (Errico et al. 2002, 2004; McDermott et al. 2003; Wharton et al. 2003). However, antibodies generated against Spastin were also reported to stain nuclei in several cell lines and mouse spinal cord neurons (Charvin et al. 2003; Errico et al. 2004). This dual subcellular localization has been proposed to reflect a role for human Spastin in processes involving highly dynamic microtubule states, such as during cell division (reflected in Spastin's nuclear localization) and in axon outgrowth, suggested by the distal cytoplasmic staining of growing axons in culture (Errico et al. 2004).

**Figure 2:**
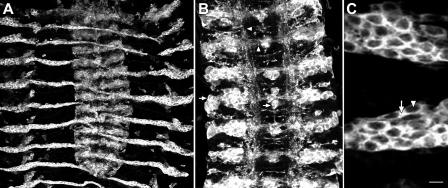
Spastin Protein Localizes to the Cytoplasm (A) In embryo “fillets” in which Spastin overexpression is driven by the *engrailed-GAL4* driver, a polyclonal antibody, pAb1239, generated against the C-terminal half of Spastin (aa 380–758) recognizes the characteristic striped pattern of Engrailed cells. Anterior is up; the CNS is the structure in the center, and the lateral epithelial stripes extend to either side. (B) An enlarged view of the CNS shows Spastin protein in these embryos localizing to neuronal cell bodies (arrow indicates the ventral unpaired midline [VUM] neurons), as well as in commissural and longitudinal axons (arrowheads). (C) A high-magnification view of the Spastin-positive epithelial cells shows that the protein fills the cytoplasm (arrow), and is excluded from the nucleus (arrowhead). Scale bar: (A) 25, (B) 10, and (C) 6.5 μm.

**Figure 7:**
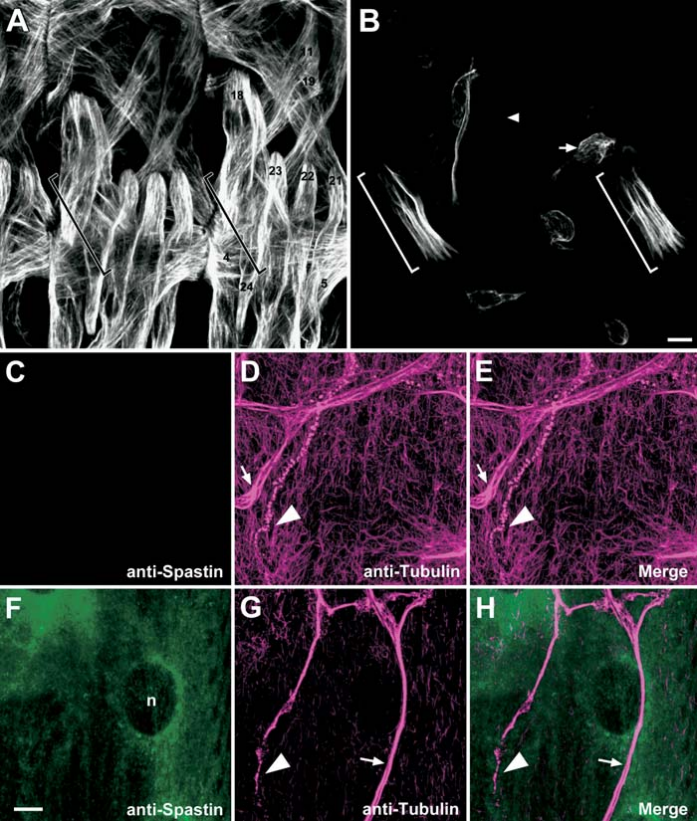
Spastin Overexpression in Muscles Erases the Microtubule Network (A) An antibody against β3-tubulin stains body wall muscles and chordotonal cap cells in stage 16 wild-type embryos. Two abdominal hemisegments are shown; muscle fiber numbers are labeled in one. The cap cells (brackets) are difficult to distinguish in this panel because of high levels of muscle tubulin staining. They extend diagonally from about the middle of muscle 18 to muscle 22. Anterior is to the left, and dorsal is up. (B) When Spastin is overexpressed in muscles (genotype: *G14-GAL4/+; T32/+*), β3-tubulin staining is very weak and has a disorganized pattern in most muscle fibers, but an intact microtubule network is still present in the cap cells, which do not express this driver (brackets). The muscle fibers are misshapen and partially (arrowhead) or completely (arrow) detached from their insertion sites. (C–H) Similarly, the microtubule network (recognized by antibodies to α-tubulin) is almost eliminated by high-level Spastin expression in third instar larval muscles. Larvae of genotype *UAS-spastin*/*MHC-GS-GAL4^213-3^; spastin^5.75^*/*TM3Ser-ActGFP* overexpress Spastin protein specifically in muscles to varying degrees. Wild-type larval muscles had undetectable levels of Spastin using pAb 1239 (C) and displayed a dense network of microtubule bundles in the muscle (D and E), as well as in trachea (D, arrow) and neurons (D, arrowhead denotes a terminal arbor). In contrast, larval muscles expressing high levels of Spastin (F) show only faint muscle microtubule staining (G and H), while tracheal (G, arrow) and neuronal (G, arrowhead) staining remain robust.

To investigate the subcellular localization of *Drosophila* Spastin, we generated a variety of antibodies against different regions of the protein (see Materials and Methods). We evaluated these antibodies by staining stage 16 embryos overexpressing Spastin in the striped Engrailed pattern from an *engrailed*-*GAL4* driver. Two different antibodies revealed Spastin expression in the expected striped pattern, which includes a subset of CNS neurons (Figure 2A). Anti-Spastin antibodies stained both the cell bodies and the axons of these neurons (Figure 2B). In the epithelial portions of the Engrailed stripes, where the cells are flat and spread out, we observed that Spastin is expressed uniformly in the cytoplasm, but did not detect any nuclear staining (Figure 2C; see also Figure 7E, showing cytoplasmic expression in muscles).

*spastin* mRNA is expressed at low levels within the embryonic ventral nerve cord (VNC) in wild-type embryos (Sun 2000; Kammermeier et al. 2003). Endogenous Spastin appears to be a very rare protein, and we have not been able to define a staining pattern in wild-type embryos or larvae that disappears in null mutants. An independently generated anti-*Drosophila* Spastin antibody was reported to stain the cytoplasm of both neurons and muscles in wild-type larvae, and staining was also detected at NMJ boutons (Trotta et al. 2004). Taken together, these results suggest that fly Spastin, like human Spastin, is likely to be a widely expressed protein that is primarily localized to the cytoplasm.

### Spastin Overexpression in Neurons Causes Collapse of the Embryonic CNS

**Figure 3:**
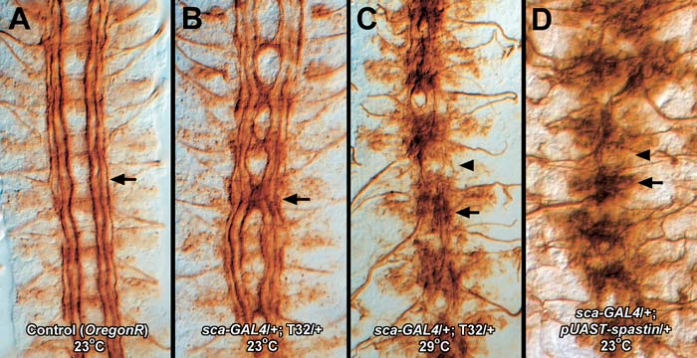
Neuronal Overexpression of Spastin Causes Midline Convergence of Embryonic CNS Axons (A) Anti-Fasciclin II (mAb 1D4) staining of filleted late stage 16 control embryos reveals three longitudinal axon bundles (arrow) on each side of the midline. Anterior is up. (B) In *sca-GAL4/+; T32/+* embryos raised at room temperature, overexpression of Spastin in neurons causes the ladder to constrict toward the midline (e.g., arrow). (C) Increased Spastin expression at 29 °C causes collapse of the CNS onto the midline (arrow). Longitudinal axon tracts are thin or absent (arrowhead). (D) A phenotype similar to that in (C) is produced by *sca-GAL4*-driven expression of the *UAS-spastin* cDNA insertion at 23 °C. Arrow and arrowhead indicate same as in (C).

Crossing the *T32* insertion line to *scabrous* *(sca)*-*GAL4,* which is expressed in neuronal precursors and neurons (Klaes et al. 1994), produced very strong CNS phenotypes. In Figure 3, these are visualized by staining with monoclonal antibody (mAb) 1D4 (Van Vactor et al. 1993), which labels a set of three longitudinal axon bundles. At 23 °C, embryos displayed abnormal midline crossing of the inner 1D4 bundle, and the entire VNC was narrowed at these crossing sites (Figure 3B). When crosses were performed at 29 °C, a temperature at which GAL4 transactivation is stronger, the VNC collapsed onto the midline and discrete longitudinal bundles were no longer apparent (Figure 3C). We also made transgenic lines bearing a full-length *spastin* cDNA driven by a UAS-containing promoter. After crossing to *sca*-*GAL4,* such lines produced even stronger phenotypes, in which the VNC collapsed at 23 °C (Figure 3D). Consistent with this observation, we found that more Spastin protein was made in driver × *UAS-spastin* embryos than in driver × *T32* insertion embryos.

### *spastin* LOF Mutations Produce Larval NMJ Phenotypes

To evaluate Spastin's functions during development, we generated several deletion mutations from the *T32* insertion by imprecise excision. We mapped their breakpoints by sequencing, and these data are displayed in Figure 1B. In line 10-12, about half of the first exon is deleted. The 17-7 deletion ends within the second intron and thus removes the entire first exon (encoding sequence up to aa 251). In both of these lines, DNA encoding the protein region conserved between human and *Drosophila* Spastin is still present. Deletion 5.75 removes the entire *spastin* gene, as well as the intergenic region and 129 bp at the 3′ end of the sequence of the adjacent predicted gene *Rox8*. Rox8 contains RRM RNA-binding domains. The 5.75 deletion removes the C-terminal 43 aa of the Rox8 protein, but does not delete into the RRM domains. The function of Rox8 is unknown, and there are no existing *Rox8* mutations (Brand and Bourbon 1993). Because the only null *spastin* mutation also affects *Rox8,* we relied on rescue experiments (see below) to demonstrate that the phenotypes we describe for the null mutant are due to loss of Spastin.

Flies homozygous for *spastin^10-12^* and *spastin^17-7^* have behavioral phenotypes, but they eclose at normal frequencies and are fertile (see below). In contrast, most homozygous *spastin^5.75^* pupae do not eclose. *spastin^5.75^* adults have very severe behavioral phenotypes, and both sexes are sterile. These results suggest that the *10-12* and *17-7* alleles are hypomorphic, and that the *spastin^5.75^* phenotype represents the null condition. RT-PCR analysis of cDNA from *spastin^10-12^* and *spastin^17.7^* animals indicated that low levels of truncated *spastin* transcripts are still produced (data not shown). These may direct synthesis of proteins initiated from internal ATGs that could retain partial function, since they include the entire conserved AAA domain.

**Figure 4:**
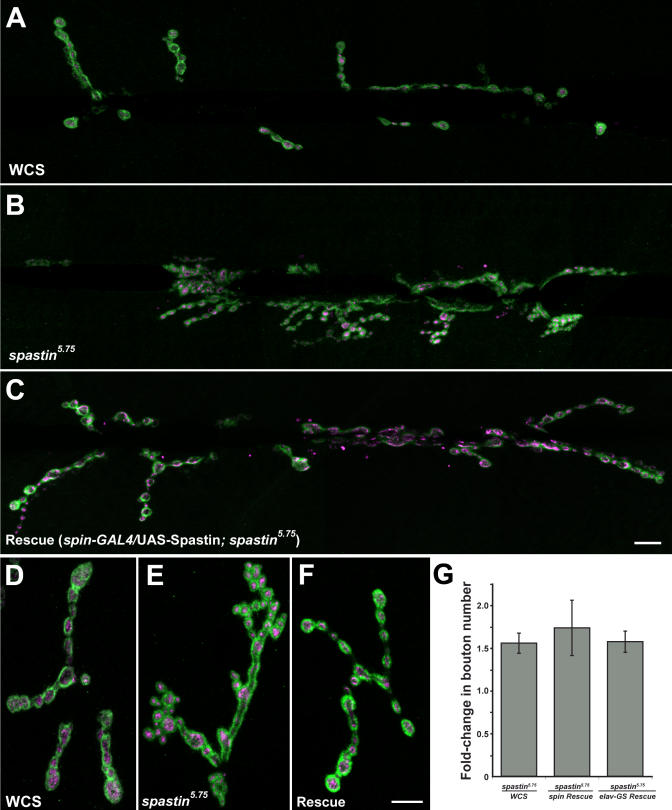
Synaptic Boutons Are Smaller, More Numerous, and Clustered in *spastin* LOF Mutants (A–F) Representative A3 NMJs on muscles 6/7 (A–C) or muscle 4 (D–F) stained with antibodies against Dlg (green) and Syt (magenta) are shown for control larvae (*WCS;* A and D), *spastin^5.75^* larvae (B and E), and larvae expressing Spastin from the *spin-GAL4* driver in a *spastin^5.75^* mutant background (Rescue; C and F). Boutons are arranged in a linear pattern in *WCS* larvae, whereas in *spastin^5.75^* larvae their distribution is more clustered and individual boutons are smaller. These phenotypes are rescued by Spastin expression via the *spin-GAL4* driver. Scale bars, 10 μm. (G) Quantitation of bouton numbers in *spastin* mutants relative to wild-type and rescued larvae demonstrates complete rescue of the null phenotype by *spin*- or *Elav*-*GS*-*GAL4*-driven expression of Spastin. *spastin*-null mutants have on average 1.6-fold more type Ib boutons on muscle 4 compared to *WCS* control larvae. Similarly, *spastin*-null mutants (of genotype *spin-GAL4/CyOKr-GFP; spastin^5.75^*) have 1.7-fold more boutons compared to their sibling rescued larvae (genotype *spin-GAL4/UAS-spastin; spastin^5.75^*). Boutons are also 1.6-fold more numerous in *spastin*-null larvae from a neuronal rescue cross (genotype +/*CyOKr-GFP; Elav*-*GS*-*GAL4*,*spastin^5.75^*/*spastin^5.75^*) compared to their siblings in which *UAS-spastin* is expressed in neurons postembryonically (genotype *UAS-spastin* /*CyOKr-GFP; Elav-GS-GAL4, spastin^5.75^*/*spastin^5.75^*).

We could not detect anatomical phenotypes in embryos homozygous for any of the *spastin* mutations. However, we saw striking morphological changes in the NMJs of *spastin^5.75^* third instar larvae. In Figure 4, the two predominant types of glutamatergic boutons at the NMJs, Ib (big) and Is (small), are visualized by double-staining larval fillets with antibodies against Synaptotagmin (Syt, magenta; Menon and Zinn 1998) and Discs-large (Dlg, green; Woods and Bryant 1991). Syt is a presynaptic protein involved in neurotransmitter release that is localized to boutons, while Dlg is a primarily postsynaptic scaffold protein localized to the subsynaptic reticulum that surrounds each bouton (Littleton et al. 1993; Lahey et al. 1994). Figure 4A and 4B show that NMJ boutons are smaller and more numerous at the muscle 6/7 NMJ of *spastin^5.75^* larvae than in *Canton S* *w−* (*WCS*) control larvae. (*WCS* was chosen as a control because, like the lines used to generate our *EP* insertion mutants, it is derived from a *Canton S* wild-type background, but it is also *w−,* like the *T32* excision derivatives. *WCS* is also commonly used for behavioral experiments.) Other NMJs are affected in a similar manner (e.g., Figure 4D–4F, showing muscle 4 synapses). The sizes of muscle fibers are normal in *spastin* mutants.

To quantify the NMJ phenotype, we counted the numbers of boutons at the muscle 4 NMJs of segments A2 and A3, where boutons typically form on the internal surface of the muscle and are thus easily imaged. Dlg is expressed at much higher levels at Ib compared to Is boutons, allowing the two types of boutons to be distinguished and counted. Because of the greater variability in Is bouton number between NMJs, we focused our quantitative analysis on the type Ib boutons. However, the numbers of both bouton types were similarly affected in *spastin^5.75^* larvae.

The number of Ib boutons per muscle 4 NMJ was increased by 1.6-fold relative to *WCS* in *spastin* mutants at room temperature (approximately 23 °C) (Figure 4G), and the boutons often formed dense clusters, particularly at the ends of NMJ branches (Figure 4E). This morphology was rarely observed in wild-type muscle 4 NMJs, where boutons were arranged more linearly (Figure 4D). The clustered boutons resemble the “satellite” boutons described by other investigators (Torroja et al. 1999; Franco et al. 2004; Koh et al. 2004; Marie et al. 2004). Hypomorphic *spastin^10-12^* and *spastin^17-7^* mutants had bouton numbers that did not differ significantly from controls.

To confirm that loss of Spastin produced the observed NMJ alterations, and to determine whether Spastin is required presynaptically or postsynaptically, we needed to evaluate rescue of the phenotype by expression of Spastin from a *UAS-spastin* cDNA insertion. This was difficult because of the early lethality produced by expression of Spastin from most drivers. *UAS-spastin* animals bearing pan-neuronal *(Elav-GAL4),* motoneuronal *(OK6-GAL4),* or pan-muscle *(24B-GAL4* or *G14*-*GAL4)* drivers did not survive to larval stages at 23 °C, and few larvae appeared even at 18 °C. However, third instar larvae in which Spastin expression from the cDNA was conferred by *spinster* *(spin)*-*GAL4,* a weak driver that functions in both neurons and muscles (Sweeney and Davis 2002), did survive at 23 °C. We were also able to obtain larvae in which Spastin expression was induced in neurons postembryonically. This was done by crossing *UAS*-*spastin* to *Elav-GeneSwitch (GS)-GAL4,* a driver line bearing a neuronally expressed GAL4 derivative that is only active in the presence of the progesterone analog RU486 (Osterwalder et al. 2001; McGuire et al. 2004). Newly hatched larvae from this cross were maintained on RU486-containing food until the third instar stage.

To assay rescue, we combined the *spin-GAL4* and *Elav-GS-GAL4* drivers and *UAS-spastin* insertions separately with *spastin^5.75^,* crossed the driver and *UAS-spastin* lines together, and assayed NMJ phenotypes in the F1 *driver-GAL4/UAS-spastin;* *spastin^5.75^* larvae. In each cross, we compared the rescued larvae to their unrescued *spastin* mutant siblings *(driver-GAL4; spastin^5.75^),* because the presence of the driver chromosome had effects on the absolute number of Ib boutons (see Materials and Methods). We observed that the ratio of muscle 4 Ib bouton numbers in *spastin* mutant controls versus rescued larvae was 1.7 for *spin-GAL4,* and 1.6 for *Elav-GS-GAL4* (Figure 4G). Since the ratio of bouton numbers for *spastin^5.75^* versus*WCS* was 1.6, this indicated that rescue was essentially complete in both cases. We also observed that the abnormal bouton clustering was eliminated in rescued larvae using either driver (Figures 4E and S1). These results demonstrate that loss of Spastin from neurons during larval development causes the NMJ bouton phenotypes seen in *spastin^5.75^* mutants.

To examine the consequences of driver-dependent postembryonic neuronal expression of Spastin in a wild-type background, we also counted boutons in *UAS-spastin; Elav-GS-GAL4* larvae grown on RU486 food (see Materials and Methods). We observed that these larvae had fewer boutons than their siblings (0.83 ± 0.07 fold change), and some of their boutons appeared larger (Figure S1C). This phenotype is very mild, but it does suggest that loss and increased expression of Spastin can produce opposite effects on the NMJ.

### Neurotransmitter Release Is Impaired in s*pastin* Mutants

**Figure 5:**
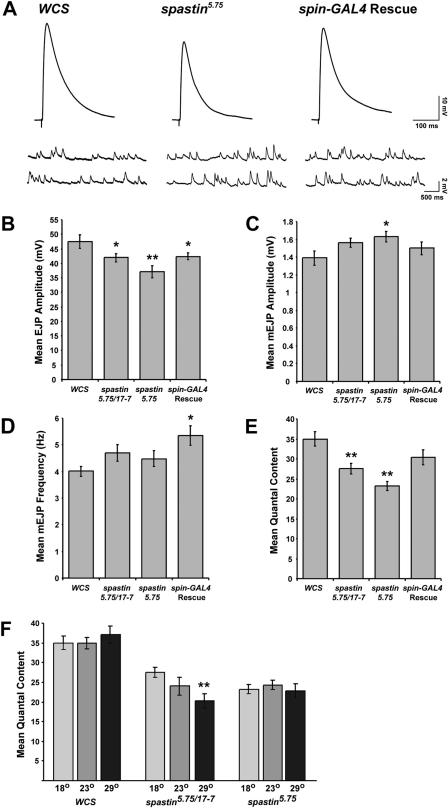
NMJs in *spastin* Mutant Larvae Display Reduced QC (A) Representative EJP (upper) and mEJP (lower) traces are shown for control *(WCS), spastin*-null mutant *(spastin^5.75^),* and *spin-GAL4/UAS-spastin; spastin^5.75^* (Rescue) larvae. All recordings were from the A3 or A4 muscle 6 NMJ. (B) The average EJP amplitude is decreased by about 20% in *spastin*-null mutants (37 ± 2.0 mV, *n =* 26) relative to control (48 ± 2.4 mV, *n =* 14) and Rescue (42.4 ± 1.2 mV, *n =* 28) larvae, and is intermediate between control and null levels in hypomorphic *spastin^5.75/17-7^* transheterozygotes (41.9 ± 1.5 mV, *n =* 22). (C) The average amplitude of spontaneous events (mEJPs) is increased slightly in *spastin* nulls relative to control and Rescue larvae. (D) The average frequency of spontaneous events is not affected in *spastin* mutants compared to control. Rescue larvae had a slightly higher mEJP frequency. (E) Average QC, a measure of the amount of neurotransmitter released per action potential, is significantly lower in transheterozygotes (28 ± 1.3) versus control (35 ± 1.7), and reduced even further in *spastin* nulls (23 ± 1.2). This decrease is completely rescued by *spin-GAL4*-driven rescue (30 ± 1.9, *p* = 0.1 compared to *WCS*). (F) Average QC is temperature dependent in *spastin^5.75^/spastin^17-7^* transheterozygous larvae, but not in homozygous *spastin-*null or control larvae. QC measured in transheterozygotes raised at 18 °C (light gray bars) is intermediate between that of control and nulls. At room temperature (dark gray) and 29 °C (black bars), similar QC values are measured in transheterozygotes and null mutants. *, *p <* 0.05; **, *p <* 0.005.

To evaluate whether *spastin* mutations cause alterations in the electrophysiological properties of the NMJ, we evaluated synaptic transmission at the muscle 6 NMJ in *WCS,* mutant, and rescued larvae raised at 18 °C. In *spastin^5.75^* larvae, there was a reduction in the amplitudes of evoked responses (excitatory junction potentials [EJPs]) to depolarization of the innervating nerves. Average EJP amplitudes in the null were reduced to 78% of the levels in control *(WCS)* larvae (Figure 5A and 5B; *p <* 0.003). We also examined the average amplitude and frequency of responses to single vesicles of spontaneously released neurotransmitter (“mini” EJPs [mEJPs]). mEJP amplitude was increased slightly, to 117% of *WCS* levels (Figure 5A and 5C; *p <* 0.03). There was no significant change in mEJP frequency (Figure 5D; *p* = 0.3).

Quantal content (QC), a measure of the number of vesicles released per evoked event, was calculated by dividing the EJP amplitude by the average mEJP amplitude. Because the evoked EJP was decreased and the mEJP increased in *spastin^5.75^* mutants, QC was reduced to 67% of *WCS* levels in these larvae (Figure 5E; *p <* 3 × 10^−6^). *spastin^17-7^/spastin^5.75^* larvae had EJP amplitude, mEJP amplitude, and QC values intermediate between those of *spastin*-null and control larvae. QC in these transheterozygotes was also decreased significantly, to 78% of *WCS* levels (Figure 5E; *p <* 0.002). The changes in EJP amplitude and QC observed in *spastin^5.75^* mutants were completely rescued by *spin-GAL4*-driven Spastin expression. Average QC in rescued larvae was not significantly different from wild-type (*p >* 0.1), and was 30% greater than in *spastin^5.75^* mutants (*p <* 0.005; Figure 5E).

We also observed that the electrophysiological phenotypes of the hypomorphic *spastin^17-7^/spastin^5.75^* larvae were temperature sensitive. While the average QC in transheterozygotes was reduced to 78% of controls at 18 °C, this effect was exacerbated at higher temperatures. At 29 °C, QC was 54% of wild-type (Figure 5F). However, QC in control or in *spastin^5.75^* null larvae was unaffected by temperature. These results suggest that the N-terminally truncated Spastin protein that is probably made from the *spastin^17-7^* allele is temperature sensitive. At 29 °C, this hypomorphic allele behaves as a null with respect to NMJ electrophysiology. Finally, we examined escaper larvae (*Elav-GAL4/+; T32/+*) overexpressing Spastin in neurons, and found that synaptic transmission in these larvae was not significantly different from wild-type (data not shown).

### *spastin* Mutant Adults Have Severe Behavioral Phenotypes

We observed that only approximately 20% of homozygous *spastin^5.75^* pupae were able to eclose at room temperature compared to 94% of heterozygotes, and the adults that emerged had severe movement defects (see Video S1). They could not fly at all, and did not even appear to move their wings, although the wings inflated and straightened in a normal manner immediately after eclosion. They also did not jump spontaneously, but would jump if persistently prodded in the abdomen. Their legs were weak: when standing, the metathoracic legs often slipped out from underneath them, and during walking they often dragged these legs (see Video S1). They also had difficulty holding on to surfaces when they were upside down. These phenotypes were temperature dependent. Null mutant flies that developed at 18 °C eclosed at much higher rates (56%) than at higher temperatures and moved more normally. Flies homozygous for the hypomorphic mutations, *spastin^10-12^* and *spastin^17-7^,* eclosed at normal frequencies at all temperatures.

**Figure 6:**
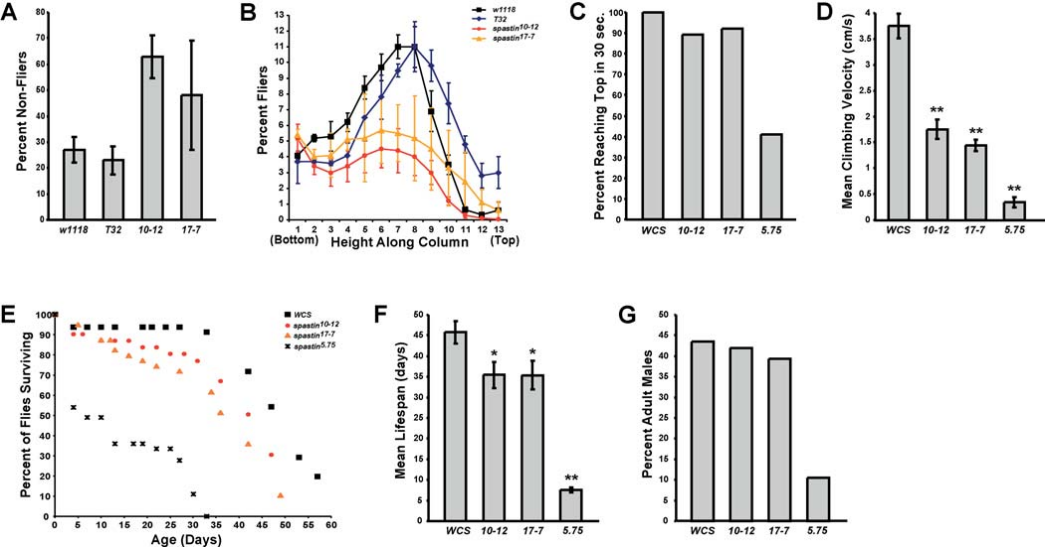
*spastin* Mutant Flies Have Compromised Motor Behavior and Reduced Lifespans (A) In a flight test assay, over twice as many adult *spastin* hypomorphs *(spastin^10-12^* and *spastin^17-7^)* fail to fly before falling to the bottom of a cylinder, in comparison to *w^1118^* and *T32* homozygous controls. (B) Although fewer than half of the hypomorphs fly, compared to more than 70% of controls, the distribution of collision sites of the fliers along the height of the cylinder parallels that of the controls, suggesting that these *spastin* mutations affect flying ability in some animals but not in others. (C and D) *spastin* mutants are compromised in their climbing ability. (C) All control *(WCS)* and nearly all *spastin* hypomorphs climb to the top of a vial in 30 s, but only 40% of *spastin* nulls do so. (D) Climbing velocity (measured for those flies that reach the top in 30 s) is 3.8 ± 0.2 cm/s in *WCS* (*n =* 45), but only 1.8 ± 0.2 and 1.4 ± 1.1 cm/s in *spastin^10-12^* (*n =* 28) and *spastin^17-7^* (*n =* 38) flies, respectively, and 0.3 ± 0.1 cm/s in *spastin^5.75^* null mutants (*n =* 17; *p <* 1 × 10^−8^ for all relative to *WCS*). (E) Lifespan curves. The curve inflection point at which *WCS* and hypomorph flies begin to die off at a rapid rate occurs at 30–35 d after eclosion, and more than 70% of hypomorph flies and 95% of wild-type flies are still alive at 30 d. In contrast, approximately 45% of *spastin^5.75^* null mutant flies die prior to 4 d after eclosion. However, the majority of the remaining null flies survive more than 25 d, so that the curve inflection point for nulls occurs only a few days before that for controls and hypomorphs. (F) Mean lifespan is 46 ± 2.7 d in *WCS* controls (*n =* 39) compared to 35 ± 3.2 and 35 ± 3.4 d, respectively, in *spastin^10-12^* (*n =* 24, *p <* 0.02) and *spastin^17-7^* (*n =* 32, *p <* 0.006), and 7.6 ± 0.6 d in nulls (*n =* 62, *p <* 10^−31^). (G) Only about 10% of *spastin^5.75^* flies eclosing at room temperature are males, while 40%–45% are males for controls and hypomorphs.

To evaluate these movement defects, we assayed flight and climbing ability in *spastin*-null and hypomorph flies (Figure 6). The flight assay could only be used for hypomorphs since null mutants were flightless. In this assay, flies were released into the top of a vertical cylinder that had been coated on the inside with oil (Benzer 1973; Atkinson et al. 2000). Poor fliers who took longer to fly fell to the bottom or collided with the lower walls of the cylinder, while good fliers who responded rapidly to being dropped collided with the upper walls. A histogram of the distribution of oil-trapped flies along the height of the cylinder showed that more than half of the *spastin* hypomorphs did not fly in time to avoid falling to the bottom of the cylinder (Figure 6A). In contrast, the majority (approximately 75%) of the controls, including *w^1118^* (another *Canton S*-derived *w−* control) flies and flies homozygous for *T32* (in the absence of a *GAL4* driver; these have orange eyes), flew well enough to distribute themselves along the sides of the column. Interestingly, for those hypomorphs that did fly out to the sides, their distribution paralleled that of the controls, suggesting that flight responses in the column were relatively normal in this subpopulation of the mutants (Figure 6B).

In the climbing assay, flies were tested for their ability to climb up the side of a vial within a limited time period. All *WCS* and almost all homozygous *spastin^10-12^* and *spastin^17-7^* flies, but only about 40% of *spastin^5.75^* flies, climbed to the top of the vial within 30 s (Figure 6C). This difference did not reflect a loss of geotactic behavior, since *spastin^5.75^* flies were typically found at the tops of their vials after several minutes. Overall, mean climbing velocity was approximately 9-fold slower for *spastin*-null mutants than for wild-type flies, while the hypomorphs were about 2-fold slower than controls (Figure 6D).

We also measured the lifespan of the flies. Under our conditions, *WCS* flies lived an average of 46 d at 25 °C*. spastin^10-12^* and *spastin^17-7^* flies had somewhat shorter lifetimes, surviving an average of 35 d. Lifespan was dramatically reduced in *spastin^5.75^* flies, which lived an average of only 8 d (Figure 6E and 6F). Examination of mortality curves (Figure 6E), however, revealed that, as in the case of flight ability in the hypomorphs, these flies had a bimodal lifespan distribution. Only 55% of flies were still alive at 4 d post-eclosion, but most of these (37% of the total) then remained alive until about 25 d post-eclosion. After this time, they rapidly died off, and no flies remained alive more than 33 d.

Another *spastin* phenotype observed in adults was male-specific lethality (Figure 6G). For *WCS* and *spastin* hypomorphs, more than 40% of eclosed adults were males. However, only approximately 10% of eclosed *spastin^5.75^* flies were male. We do not understand the origins of this phenotype.

In summary, *spastin*-null adult flies had severely compromised movement behavior and were short-lived, while *spastin* hypomorphs displayed weaker movement and lifespan phenotypes. We also examined rescue for these behavioral phenotypes. When compared to their non-rescued siblings *(spin-GAL4/CyOKr-GFP; spastin^5.75^)* from the same cross, *spin-GAL4/UAS-spastin;* *spastin^5.75^* flies climbed better, were more coordinated, and lived longer (Figure S2; Video S1), indicating that partial rescue was achieved. These flies were still very slow, and it is clear that *spin-GAL4*-driven Spastin expression did not restore behavior to the levels characteristic of control flies such as *WCS*. However, genetic background effects made the precise efficacy of rescue achieved in this experiment difficult to determine (the non-rescued *spastin^5.75^* sibling flies bearing the driver and balancer chromosomes used in the rescue cross were much more unhealthy and slow-moving than *spastin^5.75^* flies without these chromosomes).

### Spastin Overexpression Erases Microtubule Networks In Vivo

To investigate whether *Drosophila* Spastin affects microtubule networks, we overexpressed it in embryonic muscles using the *G14*-*GAL4* or *24B-GAL4* drivers, and then visualized muscle microtubules in late stage 16 embryos with an anti-β3-tubulin antibody that preferentially stains polymerized tubulin (Buttgereit et al. 1996). In wild-type embryos, a complex network of microtubules aligned along the muscle axes was observed (Figure 7A; two segments are shown). This was clearly seen in both vertically oriented (18 and 21–24) and diagonally oriented (5,11, and 19) muscles.

When Spastin was overexpressed in muscles, the muscle microtubule network completely disappeared (Figure 7B). The muscles themselves appeared rounded, and were partially or totally detached from their insertion sites. This detachment may have been a consequence of the dissolution of the microtubule network. Oriented microtubule networks could still be seen within the cap cells of the chordotonal organs in each segment (Figure 7B, brackets); these cells did not express the *GAL4* driver and therefore did not overexpress Spastin. Dissolution of the microtubule network was therefore specific to cells in which Spastin was overexpressed.

We also overexpressed Spastin in larval muscles by crossing the muscle-specific *MHC-GS-GAL4* driver line to *UAS-spastin* flies. *MHC-GS-GAL4* is RU486-inducible, but we could not feed the larvae with RU486 as this was lethal. However, *MHC-GS-GAL4* also confers late RU486-independent expression in third instar larvae, and we were able to obtain escaper larvae from the cross and double-stain these for Spastin and α-tubulin. As shown in Figure 7C–7E, larvae that lacked detectable Spastin expression had a dense network of muscle microtubules. In contrast, *MHC-GS-GAL4/UAS-spastin* larvae that had high levels of muscle Spastin displayed a dramatic reduction in microtubules, so that only faint and sparse microtubules were observed in the muscle fibers (Figure 7F–7H). The strongly staining microtubules still visible in these larvae are those of the neurons and tracheae, which do not overexpress Spastin (Figure 7G–7H). These results also show that this α-tubulin antibody preferentially recognizes polymerized tubulin, since the total amount of tubulin dimers would be the same in both sets of muscles (tubulin dimers are very stable proteins and are unlikely to be proteolyzed after severing).

**Figure 8:**
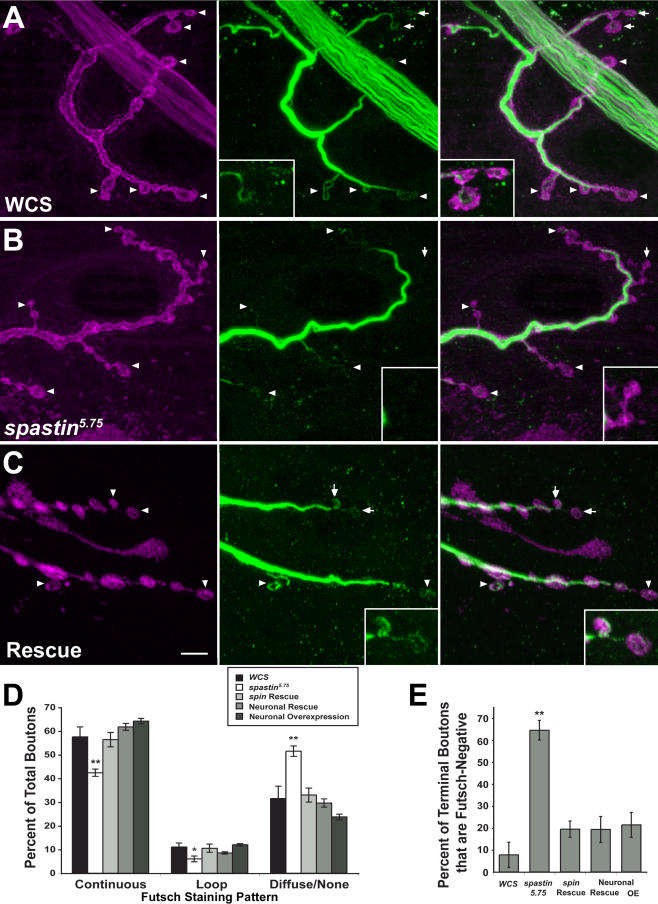
The Distribution of Stable NMJ Microtubule Bundles Marked by the MAP1B-like Protein Futsch Is Altered in *spastin* Mutant Larvae (A–C) Anti-Futsch labels stable microtubule bundles in axons, NMJ boutons, and interbouton regions. The muscle 4 NMJs in segment A3 of third instar wild-type (A), *spastin^5.75^* (B), and *spin-GAL4/UAS-spastin; spastin^5.75^* (Rescue) (C) larvae were immunostained with anti-HRP (A and B) or anti-Syt antibodies (C) to label presynaptic boutons (magenta), and mAb 22C10 to label Futsch protein (A–C, green). Arrows and arrowheads mark the terminal boutons (those at the ends of synaptic branches); boutons marked by arrows in (A–C) are enlarged in insets in the middle and right panels to show examples of Futsch patterns. (A) In control (*WCS*) larvae, terminal boutons have both looped (arrowheads) and diffuse, punctate (right panel, arrows and inset) patterns of Futsch staining. (B) In *spastin* mutants, Futsch staining appears similarly strong in axon bundles (not shown) and along the main branches of the bouton arbor. More distal and terminal boutons, however, have diffuse or no Futsch staining (arrows and arrowheads). Note the absence of green staining in insets. (C) The distribution of Futsch staining is restored to the control pattern by *spin-GAL4*-driven expression of Spastin in the mutant background (arrows and arrowheads indicate loops). Scale bar, 5 μm. (D and E) Quantitative assessment of Futsch staining data. Futsch staining at A2 and A3 muscle 4 NMJs was classified as continuous (bundles or splayed bundles), looped, or diffuse or undetectable (none) for each bouton. (D) The percentage of boutons exhibiting continuous or looped Futsch staining (relative to the total number of boutons for each NMJ) is decreased in *spastin* mutants relative to controls, while the percentage of boutons having diffuse or no staining is increased. In total, 58% ± 4.2% of boutons in controls have a continuous pattern of Futsch staining, while only 42% ± 1.5% do in mutants. Boutons in this class are predominantly along the major (more proximal) branches of the axon arbor. Similarly, 11% ± 1.7% of wild-type boutons have Futsch loops, but only 6.0% ± 1.1% do in mutants. Most mutant boutons show only diffuse or no Futsch staining (52% ± 2.2%, versus 32% ± 5.2% in controls). Futsch distribution is restored to the control pattern by *spin-GAL4*- or *Elav-GS-GAL4-*driven expression of Spastin. (E) The difference in Futsch distribution is most pronounced at terminal boutons. There is no detectable Futsch staining in the majority of terminal boutons (65% ± 4.5%) in *spastin* mutants, compared to only 7.8% ± 5.8% of terminal boutons in wild-type larvae (*p <* 2 × 10^−6^). Futsch staining is restored in most terminal boutons of *spin-GAL4*- or *Elav-GS-GAL4-*rescued larvae, with only 20% ± 3.7% and 19% ± 5.9% of boutons, respectively, showing no staining (*p* = 0.09 compared to *WCS*). Terminal bouton staining in larvae overexpressing Spastin in neurons was unaffected relative to controls (*p* = 0.12). **, *p <* 0.005; *, *p <* 0.03 relative to *WCS;* *n* >8 NMJs scored in all cases.

Interestingly, when we overexpressed Spastin in the embryonic or larval CNS, we did not observe an obvious alteration of the axonal microtubule architecture (see also Figure 8). This suggests that Spastin may be unable to disassemble stable axonal microtubule bundles. Nevertheless, the dramatic effects of Spastin overexpression on muscle microtubules suggest that the embryonic CNS collapse phenotype conferred by neuronal overexpression (see Figure 3) may also arise from breakdown of key neuronal microtubules during the axonal growth phase.

### Microtubule Bundles Are Depleted in Distal NMJ Boutons of *spastin* LOF Mutants

The finding that Spastin overexpression erases the microtubule network in muscles suggested that the *spastin* LOF NMJ phenotypes could arise from alterations in microtubule networks. To investigate this, we first examined the distribution of Futsch, a microtubule-associated protein related to vertebrate MAP1B (Hummel et al. 2000; Roos et al. 2000). Futsch staining is restricted to stable neuronal microtubule bundles. Because Futsch is not expressed in the underlying muscle, Futsch antibody staining provides the optimal method for quantitatively evaluating stable microtubules within NMJ boutons.

At muscle 4 NMJs in wild-type larvae (Figure 8A) we observed continuous microtubule bundles stained by anti-Futsch (mAb 22C10; green) within axons and along the axis of each branch of the NMJ (delineated by anti-HRP, which labels neuronal membranes; magenta). The intensity of Futsch staining weakens in the distal portions of the branches. Consistent with earlier findings, we also observed distinctive “loops” of Futsch staining within some boutons (Roos et al. 2000; Packard et al. 2002; Pennetta et al. 2002). Loops were typically observed in terminal boutons at the ends of branches. In some terminal boutons, however, we detected only punctate staining or no staining at all. This last case may reflect the limits of detection rather than the complete absence of Futsch protein in a bouton.

We quantified Futsch distribution by dividing the patterns of Futsch staining in boutons into three classes: continuous (bundles or splayed bundles), looped, and diffuse or undetectable. In *spastin^5.75^* larvae (Figure 8B), there was a shift in the Futsch pattern toward less organized morphologies (i.e., diffuse/undetectable). At the muscle 4 NMJ of *spastin* mutant larvae, 74% and 54% as many boutons contained continuous and looped Futsch, respectively, as compared to *WCS*. In contrast, 63% more boutons in *spastin* mutants displayed only diffuse or no staining (Figure 8D). These differences were most pronounced at the distal ends of the synaptic branches (Figure 8B, arrows and arrowheads). 65% of terminal boutons in mutants had no detectable Futsch staining, as compared to 8% in *WCS* (Figure 8E; *p <* 2 × 10^−6^).

The Futsch distribution phenotypes were rescued by expression of Spastin from the *spin-GAL4* or the RU486-induced, neural-specific *Elav-GS-GAL4* drivers. Rescued larvae had Futsch staining patterns very similar to those seen in *WCS* (Figure 8C–8E). These results show that the reduction in stable synaptic microtubules seen in *spastin* LOF mutants is due to loss of Spastin from neurons during larval development. We also examined Futsch staining in *Elav-GS-GAL4/UAS-spastin* larvae grown on RU486 food, but saw no difference from the pattern in wild-type controls. Thus, stable microtubules at the NMJ do not break down when Spastin is overexpressed at the levels induced by this driver.

**Figure 9:**
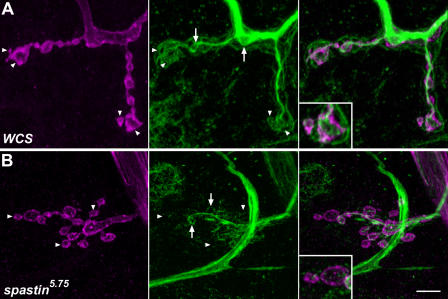
The Microtubule Network in NMJ Boutons Is Altered or Absent in *spastin* Mutant Larvae (A) In wild-type (*WCS*) larvae, an antibody against α-tubulin (green) reveals the distribution of the network of microtubule bundles within the A3 muscle 4 NMJ bouton arbor. Presynaptic bouton membranes are labeled by anti-HRP antibody (magenta). The microtubule network has a complex structure and extends into the terminal boutons (arrowheads and inset). Many proximal boutons have loops (arrows). Microtubules are also observed outside of the boundaries of the NMJ; these are within the muscle fiber, which also expresses α-tubulin. Staining of these muscle microtubules is minimized by the use of Bouin's fix. (B) In *spastin^5.75^* mutants, the microtubule network is much sparser than in controls, particularly in the distal boutons at the edges of the bouton clumps that are characteristic of *spastin* mutant NMJs (arrowheads and inset). Many of these distal boutons have little or no detectable α-tubulin staining. More proximal boutons still have tubulin loops, however (arrows). Scale bar, 5 μm.

Having demonstrated statistically significant differences in Futsch localization between control and *spastin* mutant NMJs, we then directly examined tubulin in muscle 4 NMJ boutons using fixation conditions that reduce muscle microtubule staining (see Materials and Methods). The pattern of NMJ microtubules is complex, and is difficult to quantitatively analyze because of residual signal from microtubules in the underlying muscle. However, using the α-tubulin antibody described above, we were able to clearly visualize looped microtubule structures (Figure 9A, green) within anti-HRP-labeled boutons (magenta). These loops were present both along the branches and in the terminal boutons (inset).

*spastin^5.75^* NMJs exhibited weaker α-tubulin staining than wild-type controls, particularly in terminal boutons (Figure 9B). Looped microtubule structures could be seen in some boutons in mutants. However, boutons at the ends of NMJ branches or at the outer edges of the bouton clumps that are characteristic of *spastin* NMJs often lacked any tubulin staining (inset). Thus, our results indicate that microtubule bundles are selectively depleted from the distal boutons of NMJs in larvae lacking Spastin protein.

## Discussion

Mutations in the human *spastin* gene, which encodes an AAA ATPase, are the most common cause of pure AD-HSP. We identified the *Drosophila* Spastin ortholog (see Figure 1) in a gain-of-function screen (see Figure 3). *Drosophila* Spastin is a cytoplasmic protein that can also localize to axons (see Figure 2). *spastin*-null larvae have altered NMJs in which presynaptic boutons are more numerous and smaller than in wild-type, and are organized in dense clusters (see Figure 4). These changes in bouton number and organization are rescued by expression of Spastin in neurons (see Figures 4 and S1). QC, a measure of the number of vesicles of neurotransmitter released in response to an action potential, is reduced at NMJs in both null and hypomorphic *spastin* mutants (see Figure 5).

*spastin*-null flies have severe movement defects. They cannot fly at all, and do not jump. They climb and walk very slowly, often drag their hind legs when walking (see Video S1), and have greatly reduced lifespans. *spastin* hypomorphs have milder phenotypes, displaying flying defects and a decrease in climbing speed (see Figure 6).

### Regulation of Synaptic Microtubule Networks by Spastin

The AAA domain of Spastin is quite similar to that of Katanin-60, which is a microtubule-severing protein. To determine whether Spastin might also sever or otherwise alter microtubules in vivo, we overexpressed the protein in embryonic and larval muscles. Strikingly, this overexpression erases or greatly reduces the microtubule network (see Figure 7). These data are consistent with the finding that overexpression of human Spastin in transfected mammalian cells causes microtubule disassembly (Errico et al. 2002; McDermott et al. 2003).

Having demonstrated that Spastin can cause disassembly of microtubules in vivo, we then examined how its absence affects the synaptic microtubule cytoskeleton. Based on the overexpression phenotype, one might have expected that microtubules would be more stable or more numerous in *spastin* LOF mutants. However, our observations indicate the opposite: microtubule bundles are depleted in NMJ boutons when Spastin is absent.

At the wild-type muscle 4 NMJ, boutons are arranged along linear axes. Continuous microtubule bundles run along the axes and connect to larger bundles within the innervating axon. Microtubules within boutons are typically arranged in loops and swirls. In *spastin*-null mutants, boutons are arranged in clumps, and the distal boutons of these clumps often lack any detectable tubulin staining (see Figure 9). Looped microtubule structures are present within some proximal boutons, however, and the bundles connecting the NMJ to the axon are still present. These results suggest that the absence of Spastin selectively affects the construction of the presynaptic microtubule cytoskeleton, and that the severity of the microtubule defects in a bouton are correlated with its distance from the NMJ's axonal branchpoint.

We quantitated these defects using an antibody against the microtubule-associated Futsch protein, which defines a subpopulation of stable neuronal microtubule bundles. In wild-type larvae, Futsch staining forms continuous lines along the main branches of the NMJ. Some individual boutons have Futsch loops, while others display only diffuse staining. A comparison of wild-type and *spastin*-null larvae shows that the distribution of Futsch within boutons shifts from organized structures (bundles and loops) toward diffuse patterns or the absence of detectable staining. This effect is most pronounced at terminal boutons, and is rescued by neuronal expression of Spastin (see Figure 8).

If Spastin's function in vivo is to disassemble microtubules, as suggested by our overexpression experiments (see Figure 7), why does its absence produce a paradoxical reduction in microtubules within the NMJ (see Figures 8 and 9)? One possibility is that microtubule severing is required for movement of microtubules into or within the presynaptic region. Some evidence for this idea has been published. In one study, injection of function-blocking anti-Katanin-60 antibody into cultured sympathetic neurons reduced process outgrowth, and microtubules were 4- to 5-fold longer in antibody-injected neurons than in control cells (Ahmad et al. 1999). More recent work demonstrated that expression of dominant-negative Katanin-60 reduces axonal outgrowth (Karabay et al. 2004). These results were interpreted as indicating that Katanin is required for severing microtubules to a length that allows their transport along the axon to its growing tip. When Katanin is inhibited, microtubule segments may be too long to be efficiently transported, and this results in a reduction in axon outgrowth.

Based on these findings, we suggest that the depletion of microtubules in the distal boutons of *spastin* mutant NMJs arises because severing of axonal microtubules by Spastin is necessary to generate microtubule polymers that are short enough to be efficiently moved into and through the presynaptic terminals. Perhaps Spastin normally excises sections of microtubules at branchpoints where NMJ branches leave the axon trunk, and these severed microtubule segments (or individual tubulin dimers) are then moved distally into the boutons of the NMJ as it grows.

Is Spastin also involved in axon outgrowth or guidance, as suggested by its embryonic gain-of-function phenotype (see Figure 3)? Clearly loss of Spastin activity in *Drosophila* does not strongly affect outgrowth, since the embryonic CNS axon ladder develops in a normal manner and motor axons reach their appropriate targets in *spastin* mutants. Furthermore, axonal and muscle microtubules are not detectably altered in *spastin*-null embryos. Severing of microtubules in vivo, however, may usually involve the actions of multiple severing proteins. In addition to Spastin, the *Drosophila* genome encodes three AAA ATPases whose AAA domains are closely related to that of vertebrate Katanin-60. These are Katanin-60, CG1193, and an ortholog of mammalian Fidgetins, CG3326 (see Figure 1C). None of these proteins have been genetically characterized. Of these four proteins, Spastin is most distant from vertebrate Katanin-60, yet we have shown that Spastin overexpression causes microtubule disassembly in vivo (see Figure 7). Thus, our results suggest that all four fly proteins are microtubule-severing enzymes or proteins that otherwise facilitate disassembly of microtubule networks. Perhaps each is dedicated to severing microtubules in particular cellular and subcellular contexts, and their functions may be partially redundant. If so, generation of severe phenotypes in which microtubule networks are disrupted might require loss of two or more of these AAA ATPases. In mammals, Katanin and Spastin are both expressed in CNS neurons (Wharton et al. 2003; Karabay et al. 2004), consistent with the idea that they could have overlapping functions.

After this manuscript was submitted for initial review, a paper appeared on perturbation of *Drosophila* *spastin* using transgenic RNAi techniques (Trotta et al. 2004). In direct contrast to our results, this paper concluded that (1) *spastin* is an essential gene (since crossing *spastin* RNAi flies to a ubiquitous *GAL4* driver line was reported to produce lethality), (2) *spastin* RNAi larvae have reduced NMJs and an increase in synaptic transmission, and (3) loss of Spastin from neurons produces an increase rather than a decrease in stable microtubules in the NMJ.

The conclusions in our paper are based on phenotypic analysis of *spastin* mutations that delete part or all of the coding region and on rescue of null mutant phenotypes by neuronal expression from a transgene. Our results show that *spastin* is not an essential gene: even *spastin*-null flies can eclose and live for several days, and *spastin* hypomorphs, which would be expected to more closely resemble most RNAi-perturbed flies, eclose at normal rates and have lifespans and behavior that do not greatly differ from wild-type (see Figure 6). We also determined that the *17-7* mutation, which removes more than one-third of the coding region, produces no detectable alterations in bouton number or NMJ microtubules and slightly decreases synaptic transmission, while *spastin*-null mutants have more boutons than wild-type larvae, a reduction in NMJ microtubule bundles, and more severely reduced transmission (see Figures 4, 5, 8, and 9).

In most transgenic RNAi work in *Drosophila,* different transgenic lines yield phenotypes that range from hypomorphic to near-null, and transgenic RNAi does not completely eliminate expression of the target protein (e.g., Billuart et al. 2001; Kalidas and Smith 2002). In the Trotta et al. paper, it is unclear whether more than one transgenic RNAi line was analyzed, but RNAi is described as reducing the level of Spastin protein expression by less than 4-fold. Our findings on Spastin hypomorphic phenotypes imply that such RNAi larvae would not have morphological or microtubule bouton phenotypes and that adult flies would be relatively healthy. We do not understand the origin of the discrepancies between the two sets of results.

### Implications of Studies of *Drosophila* Spastin for the Understanding of Human AD-HSP

*spastin*-null adult flies have severe movement defects. Their hind legs are particularly weak (see Video S1); this is interesting in light of the restriction of symptoms to the legs in most AD-HSP patients. Other aspects of the *spastin* mutant adult phenotypes also resemble observations made in human AD-HSP patients. The penetrance of the human spasticity phenotype is highly variable, so that some individuals carrying a *spastin* mutation appear unaffected, while others with the same mutation are confined to wheelchairs. In our experiments, we observed that some *spastin* hypomorphs exhibit normal flying behavior in a cylinder assay, while most fail to fly and crash into the cylinder base (see Figure 6A and 6B). Half of the *spastin*-null adults die within 4 d, but most of the survivors then live more than 25 d (see Figure 6E). The selective male lethality we observed (see Figure 6G) is also interesting in light of the discovery of a large *SPG4* pedigree in which only males exhibit AD-HSP phenotypes (Starling et al. 2002).

Despite these apparent parallels, there is no evidence at present that the fly behavioral phenotypes arise through mechanisms related to those that cause human AD-HSP. The anatomy of the *Drosophila* nervous system is quite different from that of the mammalian spinal cord. Furthermore, AD-HSP is thought to be a neurodegenerative disease that progresses over a period of years, and it is unclear whether neurodegeneration as a result of *spastin* mutations could occur during the short lifespan of *Drosophila*. Further work will be required to determine to what extent the *Drosophila* system can provide an organismal model for AD-HSP pathology.

The clearest implications of our work for AD-HSP emerge from the analysis of the cellular phenotypes arising from loss of Spastin. We show that the absence of Spastin alters the microtubule network at nerve terminals. Microtubule bundles are depleted in the distal boutons of the NMJ, which is a glutamatergic synapse that resembles excitatory synapses within the mammalian spinal cord. These results suggest that microtubules within the terminals of neurons in the human spinal cord could also be disordered or absent in patients with AD-HSP. The terminals of these neurons might eventually degenerate as a consequence of these microtubule defects, leading to a selective loss of distal axon segments within the spinal cord.

## Materials and Methods

### 

#### Genetics and molecular biology

For the overexpression screen, approximately 6,000 new *EP* insertion lines were generated by crossing an X chromosome *EP* line, *EP55,* to a *SbΔ2-3* transposase line. *EP* insertions on Chromosome II or III were crossed to the pan-neuronal driver line *Elav^C155^-GAL4* and the muscle driver line *24B-GAL4.* Embryos from *EP* lines that exhibited less than 20% viability in combination with either driver were immunostained with mAb 1D4. Eighteen lines had embryonic CNS and/or motor axon defects when crossed to *Elav^C155^-GAL4,* including line *T32*. The flanking genomic region of *T32* was cloned and sequenced by plasmid rescue. This sequence matched three overlapping EST's from BDGP, one of which, GH11184, contained the complete ORF of the gene downstream of the *T32* insertion. This cDNA was sequenced in its entirety. Unrooted trees for Spastin and its closest relatives were constructed using six different algorithms (fitch, kitsch, neighbor, upgma, protein maximum likelihood, and parsimony) from the Phylip package, based on alignment to the PFAM AAA consensus.

For the *spastin* excision lines, alleles *10-12, 17-7,* and *5.75* were generated via imprecise excision of *EP T32* using *SbΔ2-3*. All alleles were homozygous viable, and their deletions were mapped by PCR and sequencing of larval or adult genomic DNA. Allele *5.75* causes sterility in both sexes.

For the *spastin* rescue construct, the *UAS-spastin* cDNA construct was made by subcloning a 2.9-kb BglII fragment from GH11184 into the BglII site of pUAST (Brand and Perrimon 1993). This fragment contains the *spastin* cDNA up to 350 bp after the stop codon (excluding 681 bp of the 3′ UTR) and including 28 bp of polylinker sequence from the pOT2 plasmid at the 5′ end. The construct was injected at approximately 300 ng/μl into *KiΔ2-3* embryos and several transgenic lines recovered; experiments described here used the Chromosome II insertion line 8-3-5.

Rescue of *spastin*-null phenotypes by *spin-GAL4*-driven expression was assayed by crossing *UAS-spastin/CyOKr-GFP;* *spastin^5.75^/TM3SerAct-GFP* to *spin-GAL4/CyOKr-GFP; spastin^5.75^/TM3SerAct-GFP*. The numbers of Ib boutons in rescued larvae *(UAS-spastin/spin-GAL4; spastin^5.75^/spastin^5.75^)* were compared to those in unrescued sibling mutants *(spin-GAL4/CyOKr-GFP; spastin^5.75^/spastin^5.75^)* to calculate the ratios used to determine the efficacy of rescue (see Figure 4G). This was done because we observed that the presence of the driver chromosome in the background increased the absolute number of Ib boutons, so that the appropriate control was to the sibling mutants also bearing this chromosome. Rescue of *spastin*-null phenotypes by postembryonic *Elav-GS-GAL4*-driven expression was assayed by crossing *UAS-spastin/CyOKr-GFP;* *spastin^5.75^/TM3SerAct-GFP* to *Elav-GS-GAL4, spastin^5.75^/TM6B* and raising the larvae on RU486-containing food as described (Osterwalder et al. 2001; McGuire et al. 2004). The numbers of Ib boutons in rescued larvae *(UAS-spastin/+; Elav-GS-GAL4, spastin^5.75^/spastin^5.75^)* were compared to those in unrescued sibling mutants *(CyOKr-GFP/+; Elav-GS-GAL4, spastin^5.75^/spastin^5.75^)* as described above for *spin-GAL4* rescue. The effect of postembryonic neuronal overexpression of Spastin in a wild-type background was assayed by counting Ib bouton numbers in *UAS-spastin/+; Elav-GS-GAL4, spastin^5.75^/TM3SerAct-GFP* larvae from this same cross and comparing them to the numbers for *UAS-spastin/+; Elav-GS-GAL4, spastin^5.75^/spastin^5.75^*. The wild-type control lines used were *Oregon R,* *w^1118^,* and *WCS*, a *Canton S* line backcrossed ten times to *white* (gift of Anne Simon). For larval overexpression experiments, the *MHC-GS-GAL4^213-3^* driver was used to induce muscle-specific expression of *UAS-spastin* at 23 °C in the absence of RU486. Expression induced by this driver was confirmed in a separate cross using *UAS-EGFP*.

#### Generation of Spastin antibodies

Regions of the *spastin* cDNA encoding aa 136–416 (pGEX-T32PvuII), 1–167 (pAcG2T-T32BamRIa), and 380–758 (pAcG2T-T32BBA) were subcloned from GH11184 into bacterial (pGEX) or baculovirus (pAcG2T) expression vectors. Expressed protein (in the form of inclusion bodies for T32PvuII) was injected into guinea pigs (Covance, Princeton, New Jersey, United States) and the antiserum tested on *en-GAL4/+2/+* embryos. Antisera against T32PvuII (86EX) and T32BBA (1239) both showed strong staining in the Engrailed pattern; thus, both recognized overexpressed Spastin. 86EX was purified by incubation with membrane-bound pGEX-T32PvuII protein and subsequent elution with 100 mM glycine (pH 2.5) followed by neutralization with 3M Tris (pH 8.8). pAb1239 was affinity purified using the immunogen bound to Affi-gel10 beads (Bio-Rad Laboratories, Hercules, California, United States), followed by preabsorption with *spastin^5.75^* larval fillets.

#### Embryonic and larval immunocytochemistry

Stage 16 embryos were fixed and stained using standard methods (Patel 1994) with mAb 1D4 (1:5) or anti-β3-tubulin (1:500; a gift of R. Renkawitz-Pohl and D. Buttgereit). Third instar larvae were live-dissected in room temperature HL3 (see below) or PBS and fixed in 4% paraformaldehyde or Bouin's fix (for staining of NMJs with anti-tubulin) for 25 min. Primary antibodies used on larvae were mouse anti-Syt at 1:400 (Menon and Zinn 1998), rabbit anti-Dlg (1:2,000; a gift of D. Woods and P. Bryant), mAb 22C10 (anti-Futsch, 1:50; Developmental Studies Hybridoma Bank, Iowa City, Iowa, United States), rabbit anti-HRP (1:250, Cappel, MP Biomedicals, Irvine, California, United States), mouse anti-α-tubulin DM1A (1:500; Sigma, St. Louis, Missouri, United States). Anti-Spastin pAb 1239 was used at 1:300. Staining was visualized with HRP-conjugated goat anti-mouse secondary (1:200; Jackson Laboratory, Bar Harbor, Maine, United States) or Alexa-Fluor 488 and 568 anti-mouse, -rabbit, or -guinea-pig secondaries (1:200; Molecular Probes, Eugene, Oregon, United States). All fluorescently labeled samples were imaged by acquiring *z*-series projections with a Zeiss (Oberkochen, Germany) 510 inverted confocal microscope and 63×/1.4 n.a. or 100×/1.2 n.a. PlanApo objectives. Only larval segments A2 and A3 were analyzed. Individual boutons were defined as a Syt-positive area encircled by Dlg-positive staining (see Figure 4), or a Syt- or HRP-positive varicosity in the synaptic arbor (see Figure 8). All type Ib boutons were scored for each muscle 4 NMJ. Average bouton numbers per muscle 4 (see Figure 4) were as follows (mean ± s.e.): *WCS*, 44 ± 2.6 (*n =* 26 NMJs); *spastin^5.75^* mutant, 68 ± 2.4 (38); *spin* rescue, 50 ± 4.9 (26); mutant sibling with *spin* driver chromosome, 86 ± 4.6 (8); *Elav-GS* rescue, 70 ± 3.0 (19); and mutant sibling with *Elav-GS* driver chromosome, 110 ± 9.3 (21). *p <* 0.02 by one-way ANOVA for all paired comparisons.

#### Electrophysiology

Intracellular recordings were obtained at 18 °C, using sharp microelectrodes (boroscilicate glass, 1.0 mm OD; 18–35 MΩ resistance; World Precision Instruments Sarasota, Florida, United States) filled with 3M KCl, from body wall muscle 6 (segments A3 or A4) of filleted third instar larvae, following standard methods (Jan and Jan 1976). Larvae were bathed in HL3 solution (Stewart et al. 1994), in mM: NaCl, 70 (EM Science, Gibbstown, New Jersey, United States); KCl, 5; MgCl_2_, 20; NaHCO_3_, 10; HEPES, 5; Sucrose, 115; Trehalose, 5; and CaCl_2_, 1 (Sigma). Larvae were visualized with a 5×/0.10 n.a. Olympus (Tokyo, Japan) objective on an Olympus BX50WI microscope. EJPs were evoked by pulling the cut end of the innervating segmental nerve into a heat-polished suction electrode and passing a depolarizing pulse sufficient to depolarize both motoneurons (Grass SD9 stimulator). For each experiment, 10–15 single EJPs evoked at 0.2 Hz were recorded, and then spontaneous mEJPs recorded for 1 min afterwards. Only recordings with resting membrane potential below −60 mV were acquired. The average resting membrane potential for control *(WCS)* larvae was −72.2 mV, and did not differ significantly from any of the experimental groups. Average muscle input resistance in control larvae was 8.9 MΩ, and differed significantly only from the input resistance determined for *spastin ^5.75^*/*spastin^17-7^* transheterozygotes (7.5 MΩ *p <* 0.04). Recordings were performed using an Axon Instruments (Foster City, California, United States) Axopatch 200B amplifier with CV203BU headstage operating in current clamp mode. The signal was low-pass filtered at 5 kHz, digitized through an Axon Instruments Digidata 1322A 16-bit acquisition system, and recorded using Axon Instruments Clampex 8.2 software. Mean EJP amplitude was determined by averaging all single EJPs with Axon Instruments Clampfit 8.2, and corrected for nonlinear summation according to McLachlan and Martin (1981) and Feeney et al. (1998). mEJPs were measured using Mini Analysis Program (Synaptosoft, Decatur, Georgia, United States). mEJPs with a slow time course arising from neighboring electrically coupled muscle cells were excluded from analysis (Zhang et al. 1998). QC for a given NMJ was estimated by dividing the average EJP amplitude by the average mEJP amplitude. Statistics were calculated using one-way ANOVA.

#### Adult phenotypes

Eclosion rates were determined by counting numbers of empty versus full (dead) pupae on the sides of bottles in which flies had been allowed to lay for comparable time periods. The flight test assay was performed at room temperature using an opaque cylinder (a 52-cm-tall pipette washer, 18 cm in diameter) coated on the inside with fresh mineral oil. Flies of a given genotype were dumped through a hole in the center of a lid at the top. The cylinder was divided into bins along its height, and the number of flies per bin counted. Flies of different genotypes were age-matched; more than 200 flies were counted for each. The climbing assay was performed on 4–5 d old flies maintained individually in vials. Climbing velocity for each fly was measured by transferring it to an empty vial, banging it to the bottom, and then measuring either the time required to reach the top of the vial or the maximum distance it climbed in 30 s, whichever came first. Three trials were performed, and the best speed was used. For lifespan tests, flies were maintained at 25 °C, transferred every 3 d to fresh food vials, and their lifespan noted.

## Supporting Information

Figure S1 Spastin Expression in Neurons Rescues the *spastin* Mutant Morphology Representative muscle 4 NMJs stained with antibodies against Dlg (green) and Syt (magenta) are shown for (A) *spastin^5.75^* mutant (genotype +/*CyOKr-GFP; Elav*-*GS*-*GAL4*,*spastin^5.75^*/*spastin^5.75^*), (B) neuronally rescued *(UAS-spastin* /*CyOKr-GFP; Elav-GS-GAL4*,*spastin^5.75^*/*spastin^5.75^),* and (C) neuronally overexpressing *(UAS-spastin* /+*; Elav-GS-GAL4*,*spastin^5.75^*/*TM3Ser-ActGFP)* larvae. The clustered, smaller, and more numerous boutons observed in mutant NMJs (A, arrowhead) are absent in neuronally rescued larvae, which resemble controls (*WCS;* see Figure 4D). Spastin overexpression in neurons produces an opposite morphological phenotype compared to the loss of function: boutons appear slightly larger than in wild-type, and bouton counts show that they are reduced in number (83% of control; see text). Scale bar, 5 μm.
(1.4 MB TIF).

Figure S2 Adult Behavior Is Partially Rescued by *spin-GAL4*-Driven Expression of Spastin in the *spastin*-Null Background Behavioral tests were performed on flies from the four genotypes arising from the *spin-GAL4* rescue crosses, raised at 18 ^o^C. These genotypes were (1) *spin-GAL4/UAS-spastin;* *spastin^5.75^* (spin Rescue), (2) *spin-GAL4*/*CyOKr-GFP;* *spastin^5.75^* (non-rescued *spastin* mutant, denoted 5.75[R]), (3) *spin-GAL4/UAS-spastin;* *spastin^5.75^*/*TM3SerAct-GFP* (Cy^+^ Ctrl; heterozygous for the *spastin* mutation), and *spin-GAL4*/*CyOKr-GFP; spastin^5.75^/TM3SerAct-GFP* (Cy Ctrl; heterozygous for the *spastin* mutation).
(A) Climbing behavior. None of the *spastin* mutants (0%) from these crosses (5.75[R]; *n =* 21) reached the top of the vial in the prescribed 30 s time limit, compared to 8% for Rescue flies (*n =* 75), and 100% for both *spastin/+* controls (*n =* 39 and 21). Twenty-seven percent of mutants (5.75[R]) did not climb at all, compared to only 4% of the Rescue flies and 0% of the *spastin/+* controls. Thus, although both genotypes in the mutant background (homozygous for *spastin^5.75^*) were much weaker than either *spastin^5.75^* heterozygous control, Rescue flies showed improved climbing ability compared to the mutants.
(B) Similar to the results in (A), mean lifespan in *spastin* mutants (10 ± 1.3 d, *n =* 32) was significantly rescued by *spin*-driven expression of *spastin* (16 ± 1, *n =* 95, *p <* 0.004), although lifespans were much shorter in *spastin^5.75^* homozygotes than in heterozygous *spastin/+* controls (43 ± 3.1 and 44 ± 2.7; *n =* 20 each).
(218 KB PDF).

Video S1 Motor Behavior in Control, *spastin^5.75^* Mutant, and *spin-GAL4*-Rescued Flies Flies are shown moving in a vial.
Segment 1: Wild-type. One female and one male *WCS* fly are shown. Note the rapid rate at which they walk, as well as exhibiting climbing, jumping and flying behaviors. When still, their legs are controlled, and they are able to walk upside-down (out-of-focus fly near end of segment) for prolonged periods without falling.
Segment 2: Mutant. One *spastin^5.75^* female is shown. Leg weakness is obvious, particularly for the mesothoracic and metathoracic legs, both when walking and standing still. She climbs poorly, and when rotated so that she is upside-down, is unable to maintain a hanging position. No wing movement or jumping is observed.
Segment 3: Rescue. In *spin-GAL4/UAS-spastin;* *spastin^5.75^* flies, Spastin expression via the *spin-GAL4* driver partially rescues the movement defects seen in *spastin^5.75^* mutants. Two males are shown, followed by one female. Note their improved leg steadiness, velocity, and hanging ability. These flies can also jump spontaneously. The female appears to be less fully rescued; however, she is able to walk upside-down for prolonged periods, and exhibits wing movement.
(7.4 MB MOV).

## Abbreviations

aa: amino acid
AD-HSP: autosomal dominant hereditary spastic paraplegia
CNS: central nervous system
Dlg: Discs-large
EJP: excitatory junction potential
*GS*: *GeneSwitch*
LOF: loss of function
mAb: monoclonal antibody
mEJP: “mini” excitatory junction potential
NMJ: neuromuscular junction
QC: quantal content
*sca*: *scabrous*
*spin*: *spinster*
Syt: Synaptotagmin
VNC: ventral nerve cord
*WCS*: *Canton S* *w−*
