# Supplementary figures and images for: Drosophila Spastin Regulates Synaptic Microtubule Networks and Is Required for Normal Motor Function
Source: PLoS Biol. 2004 Nov 30;2(12):e429. doi: 10.1371/journal.pbio.0020429 (PMC532392; doi:10.1371/journal.pbio.0020429)

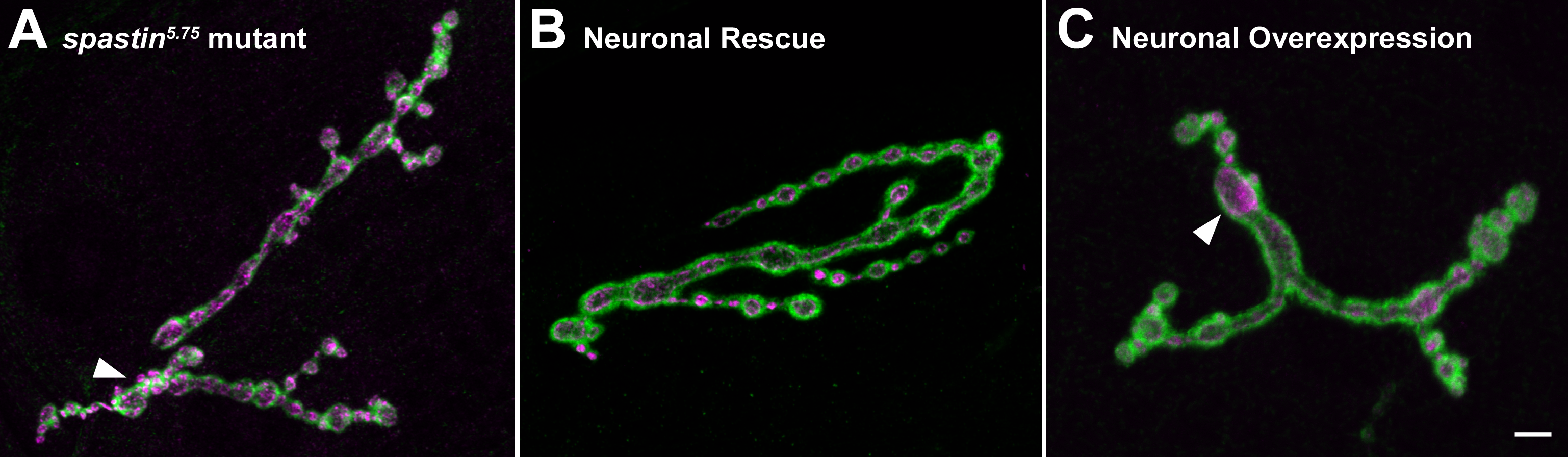

Supplement: Figure S1 — Representative muscle 4 NMJs stained with antibodies against Dlg (green) and Syt (magenta) are shown for (A) spastin5.75 mutant (genotype +/CyOKr-GFP; Elav-GS-GAL4,spastin5.75/spastin5.75), (B) neuronally rescued (UAS-spastin /CyOKr-GFP; Elav-GS-GAL4,spastin5.75/spastin5.75), and (C) neuronally overexpressing (UAS-spastin /+; Elav-GS-GAL4,spastin5.75/TM3Ser-ActGFP) larvae. The clustered, smaller, and more numerous boutons observed in mutant NMJs (A, arrowhead) are absent in neuronally rescued larvae, which resemble controls (WCS; see Figure 4D). Spastin overexpression in neurons produces an opposite morphological phenotype compared to the loss of function: boutons appear slightly larger than in wild-type, and bouton counts show that they are reduced in number (83% of control; see text). Scale bar, 5 μm. (1.4 MB TIF). [file pbio.0020429.sg001.tif]

**A**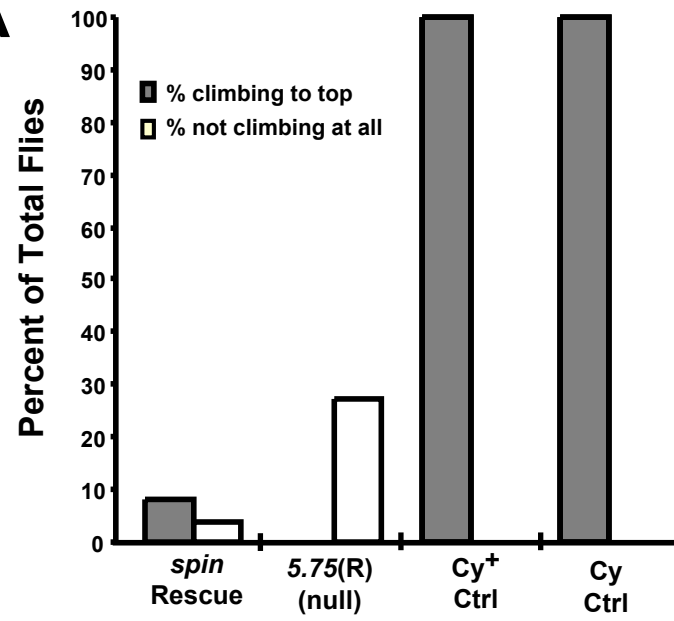**B**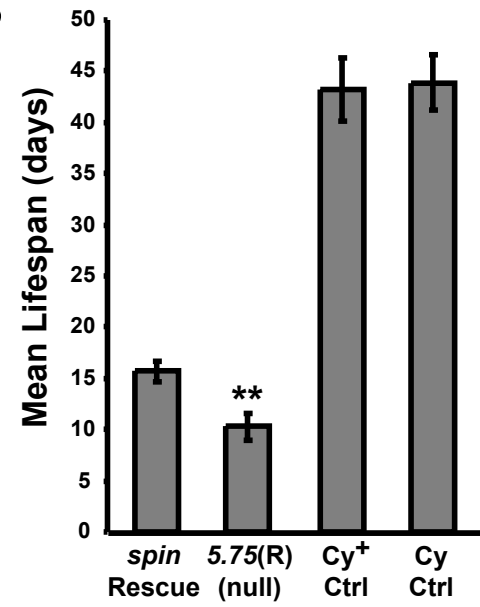

Supplement: Figure S2 — Behavioral tests were performed on flies from the four genotypes arising from the spin-GAL4 rescue crosses, raised at 18 oC. These genotypes were (1) spin-GAL4/UAS-spastin; spastin5.75 (spin Rescue), (2) spin-GAL4/CyOKr-GFP; spastin5.75 (non-rescued spastin mutant, denoted 5.75[R]), (3) spin-GAL4/UAS-spastin; spastin5.75/TM3SerAct-GFP (Cy+ Ctrl; heterozygous for the spastin mutation), and spin-GAL4/CyOKr-GFP; spastin5.75/TM3SerAct-GFP (Cy Ctrl; heterozygous for the spastin mutation). (A) Climbing behavior. None of the spastin mutants (0%) from these crosses (5.75[R]; n = 21) reached the top of the vial in the prescribed 30 s time limit, compared to 8% for Rescue flies (n = 75), and 100% for both spastin/+ controls (n = 39 and 21). Twenty-seven percent of mutants (5.75[R]) did not climb at all, compared to only 4% of the Rescue flies and 0% of the spastin/+ controls. Thus, although both genotypes in the mutant background (homozygous for spastin5.75) were much weaker than either spastin5.75 heterozygous control, Rescue flies showed improved climbing ability compared to the mutants. (B) Similar to the results in (A), mean lifespan in spastin mutants (10 ± 1.3 d, n = 32) was significantly rescued by spin-driven expression of spastin (16 ± 1, n = 95, p < 0.004), although lifespans were much shorter in spastin5.75 homozygotes than in heterozygous spastin/+ controls (43 ± 3.1 and 44 ± 2.7; n = 20 each). (218 KB PDF). [file pbio.0020429.sg002.pdf]
